# Do the labour market returns to university degrees differ between high and low achieving youth? Evidence from Australia

**DOI:** 10.1186/s12651-018-0241-0

**Published:** 2018-05-22

**Authors:** Gary N. Marks

**Affiliations:** 1 Directorate of Government, Policy and Strategy, Australian Catholic University, Level 4, 232 Victoria Parade, East Melbourne, VIC 3002 Australia; 2 Locked Bag 4115, Fitzroy MDC, Fitzroy, VIC 3065 Australia

**Keywords:** University degree, Vocational qualifications, Youth, Occupational status, earnings, unemployment, Full-time employment, I26 Returns to Education, J24 Human Capital

## Abstract

In almost all developed countries there has been substantial growth in university education over the last half-century. This growth has raised concerns that the benefits of university education are declining and that university education is not appropriate for students who, without the expansion, would not have been admitted. For such students, vocational education or direct entry to the labour market may be more appropriate. The purpose of this study is to examine the effects of university and vocational qualifications, net of other influences on a variety of labour market outcomes for Australian youths up to age 25; and if the benefits of university degrees differ across the achievement continuum. Achievement is measured by test scores in the OECD’s PISA assessments. The six labour market outcomes investigated are: occupational status, hourly and weekly earnings, employment, unemployment and full-time work. The study finds that university degrees provide substantially superior labour market outcomes which are not confined to high and average achievers, at least for this cohort in their formative years in the labour market.

## Introduction

One of the most dramatic changes in the last half-century in developed countries has been the expansion of university education, from elite to mass participation. Between the 1960 and 2005, the proportion of US high school graduates enrolling at college increased from 45% to between 65 and 70% (Alon 2009, p. 788). For Canada, McIntosh (2010, p. 458) observes that post-secondary education increased by a factor of six between 1961 and 1997. Arum et al. (2006, pp. 15, 16) show a doubling of higher education *participation* across developed countries from 20% in the 1960s to 40% in the 1990s. In most countries graduation from university—what the OECD (2009, p. 62) refers to as tertiary type A education—has increased to around an average of 40% in OECD countries with substantial increases in many countries over the relatively brief period, 2000–2007. According to the *European social survey* the proportion of graduates in youth cohorts in European countries increased, on average, from about 12% in 1950–1960 to over 40% in 2001–2010 (Koucký et al. 2010, p. 10). Participation rates are highest in Ireland, Denmark, Spain and Norway at around 60% or more.

For Australia, less than 5% of cohorts born before World War II attended university. For the 1970–1981 cohort, the figure was close to 20% (Marks and McMillan 2007, p. 362). In 2012 of those aged 25–35, 39% have a bachelor degree or higher university degree compared to 25% of those aged 55–64 and 15% of those aged 65 or more (Wilkins 2015, p. 70). For youth cohorts born in the early 1980s, 30–35% completed a university degree (Hérault and Zakirova 2015, p. 86). According to Jerrim and Vignoles (2015), 39% of the youths enroll at university by age 20, a rate similar to that for other Anglophone countries.

There is a broad political consensus to further expand university education by political parties, universities and stakeholders (Universities Australia 2013). For example, the Australian Labour Party’s platform has a target of 40% of 25–34 year-olds holding bachelor level degree or higher by 2025. The justifications for further expansion are: universities’ role in facilitating innovation and the knowledge economy, the superior labour market outcomes of university graduates and satisfying the increased demand for university education. There is also a, albeit smaller, consensus that youth unemployment and other labour market problems in the school-to-work transition are best addressed by further investments in education and training rather than politically sensitive reforms to the labour market. It is argued that vocational education and training strongly facilitates young people in the school-to-work transition and expanding it would provide substantial economic benefits in productivity, labour market participation and GDP growth (e.g. Rudd et al. 2007). For Australia, the OECD (2010) advocates both an expansion of vocational education and increased labour market flexibility. During the last decade there have been several substantial changes to the provision of vocational education (see Dempsey 2013) aiming to enhance the work skills of non-university bound youth.

There are concerns that the expansion of the university sector has gone too far. It is logical to suppose that with expansion, university degrees become devalued in the labour market. The expansion has occurred mainly by formerly non-university institutions—Centres of Advanced Education, technical, teacher and nursing colleges—being transformed into universities. Of the 25–35-year-old cohort, only 23% received their highest university qualification from one of the high status ‘group of eight’ universities. For those aged 65 and older, the comparable figure was 45% (Wilkins 2015, p. 70). Increased university participation means there are more academically weaker students at university than in comparable older cohorts. There is concern that there is already an oversupply of university graduates that cannot find appropriate employment especially in particular disciplines (Vogel 2013; Papadopoulos 2014). The labour market outcomes of recent graduates have continued to worsen (marginally) following their deterioration in the aftermath of Global Financial Crisis (Guthrie 2015).

This concern may be misplaced. Analyzing youth cohort data, Hérault and Zakirova (2015) found premiums for earnings of between 8 and 11% for completed bachelor degrees in the years following graduation. They also identified enrolment premiums of a similar magnitude which increase with time since dropping out of university. There were no completion premiums for vocational education although there were sizable enrolment effects, especially in the first year after completing a spell of post-school education. In subsequent years, the labour force benefits of enrolment in vocational education were smaller and often not statistically significant. A recent analysis of Australian youth data from several cohorts noted slightly lower unemployment rates for university degrees for both males (2.0%) and females (2.1%) compared to males (2.6%) and females (2.2%) overall (Hérault et al. 2012).

### Purpose of this study

The purpose of this study is to compare the benefits (or otherwise) of university degrees and vocational qualifications for a wider range of labour market outcomes and to examine if the labour market benefits associated with university degrees also apply to low achievers whom without the expansion of university education would not have had the opportunity to obtain a university degree. The data analyzed is from a younger Australian youth cohort than analyzed in the studies cited above.

The labour market outcomes investigated are: occupational status, hourly earnings, weekly earnings, employment, unemployment, and full-time employment. Hourly earnings allow comparison of the returns to qualifications per hour worked. Among Australian young people there is considerable part-time and casual employment. Weekly earnings are a much better indicator of the standard of living. Simply having a job is a positive labour market outcome whereas unemployment is the most undesirable outcome. A full-time job indicates a strong attachment to the labour market and is often the first step in a career.

There are a variety of other influences on youth labour market outcomes that must be considered in any analysis of the school-to-work transition. These include gender, socioeconomic background, achievement or test scores, school completion and labour force experience. Gender, qualifications and labour force experience are standard variables to include in labour force analyses. They are also important in studies of the youth labour market (Ryan 2001). Typically, separate analyses are conducted for young men and young women since there are important gender differences, notably in earnings, labour force participation and full-time work. The OECD (2010) emphasizes the importance of a ‘good start’, generally defined as appropriate full-time employment, for young people’s subsequent labour market experiences. Similarly, there is much evidence for the scarring effects of unemployment (Arulampalam et al. 2000; Gangl 2004; Gregg 2001; OECD 2010). Test scores taken at adolescence, a proxy for ability, have effects on (adult) occupational status, earnings, unemployment and other labour market outcomes (Marks 2014, pp. 91–112; Warren et al. 2002).

Analyses of labour market outcomes in Australian youth cohorts typically find effects for gender, socioeconomic background, test scores and labour force experience on earnings, unemployment and occupational group, net of educational qualifications (Huq 2014; Hérault et al. 2012; Marks and Fleming 1998a, b; Buchler and Dockery 2015; Marks 2005). Increasing school completion (that is completing year 12) has long been a policy goal and recent work suggests that school completion is beneficial (Ryan 2011).

The question of whether the effects of educational qualifications on labour market outcomes differ by student achievement is examined using students’ test scores from the OECD’s Program for International Student Assessment (PISA) tests conducted when they were 15 years of age.

## Materials and methods

### Data

In 2003, a nationally representative sample of approximately 12,500 students aged 15 years was selected to participate in PISA, conducted by the OECD. The PISA sample was constructed by randomly selecting 50 students aged 15 years from each school from a sample of schools designed to represent all states and sectors. Assessments in mathematical literacy, reading literacy, scientific literacy and problem-solving were administered in their schools to provide information on student achievement. Students also completed a background questionnaire about their families, educational and vocational plans, attitudes to school and a range of other matters. In a follow-up telephone interview, students provided further school and work information. Of the 12,500 students in the PISA sample, 10,370 were successfully contacted to participate in the subsequent 2003 Longitudinal Studies of Australian Youth study. This was the basis for a subsequent longitudinal survey of Australian youth (LSAY-Y03) on school-to-work transitions. From 2004 until 2013, cohort members were interviewed annually using computer-assisted telephone interviews and in 2012 and 2013, respondents also had the option to complete their interviews online (NCVER 2010b).

The original PISA sample design over-sampled small states, non-government schools and Indigenous students. Larger schools had a greater chance of selection than smaller and very small schools were excluded. Sample attrition between waves compounded each year, so that the sample size in the final 2013 wave was only 3741, only 36% of the 10,370 respondents in the original LSAY Y03 sample. There was differential attrition with attrition more common among respondents with parents with lower status occupations and less education, respondents from single parent families and especially respondents with lower achievement scores (Lim 2011, pp. 14, 15). The weights were calculated for each wave adjusting for sampling probabilities and attrition (Lim 2011). The estimates presented in this paper are based on the final weights and can be considered as the best estimates of population parameters.

The major dilemma in analysing initial youth labour market outcomes with longitudinal data is that in the early years most respondents are in full-time education so too few are in the labour market. In 2004, 90% were in full study and in 2008, the mid-point of the study, 50% were in full-time study. In later years, a much higher proportion were in the labour market but the sample size is considerably smaller due to attrition. Analyzing data from one or from a selection of calendar years will necessarily discard useful data. This problem is overcome by focusing on observations rather than respondents, so that all appropriate respondent observations are utilized. The data were converted to a person-year data set so that for each respondent there are between one and ten observations of their education and labour market characteristics.

### Measures

Most of the measures used in this study were constructed from the derived variables already in the data set. The derived variables are detailed in a LSAY technical paper (NCVER 2010a).

#### Labour market outcomes (dependent variables)

The measures of *occupation status* are based on respondents’ occupations for each year which were coded to the four-digit 2006 Australian and New Zealand standard classification of occupations (ANZSCO) by the Australian Bureau of Statistics. They were then converted to the Australian Socioeconomic Index known as AUSEI06 (McMillan et al. 2009). Conceptually, this measure and similar socioeconomic indices are based on the idea that occupations convert human capital (education) into material rewards (income). Ganzeboom et al. (1992) detail the conceptual basis and procedure. AUSEI06 scores were calculated iteratively for each of the 358 four-digit ANZSCO occupational codes to maximize the effect of occupational status as an intervening variable between education and income, net of education. AUSEI06 scores were rescaled to range between zero and one hundred. Indicative scores are 100 for medical practitioners and judges, 85 for school teachers, 63 for computer technicians, 25 for motor vehicle mechanics and 19 for bakers.

*Hourly* and *weekly earnings* were constructed from the respective derived variables. The variables were converted into 2013 dollars adjusted for inflation by the consumer price index (ABS 2015). Earnings data were obtained from both employees and the self-employed.

The measures of *employment*, *unemployment* and *full*-*time employment* were constructed from the derived variables for labour force status and full-time or part-time employment. For each year, three dichotomous variables were constructed distinguishing employed (scored one) from not-being-employed (scored zero); unemployed (scored one) versus employed (scored zero); and employed full-time (scored one) versus not-employed-full-time, that is employed part-time or employed under some other arrangement (scored zero).

#### Predictor variables

*Gender* was measured by a dichotomous dummy variable, young men coded one and young women coded zero.

Student achievement is measured by students’ combined scores in the 2003 PISA tests in reading, mathematical and scientific literacy. In the 2003 PISA round all students were tested in mathematics and due to the rotated design about half were tested in reading, science or problem solving (OECD 2004, p. 336). Student performance in each domain was measured by five plausible values. The plausible values are the result of the rotated design and Item Response Theory methodology. The measures of student performance have been ‘conditioned’ to reduce measurement error increasing the correlations across domains to between 0.8 and 0.9 (Bond and Fox 2001, p. 259; Cromley 2009). The mean of the students’ plausible values in mathematics, reading, science and problem solving were used to construct a single variable measure of test score, which was subsequently standardized to a mean of zero and a standard deviation of one.

Achievement tests such as PISA are cognitively demanding and are often understood as measures of ability. In PISA, literacy is defined generally as “concerned with the capacity of students to apply knowledge and skills in key subject areas and to analyze, reason and communicate effectively as they pose, solve and interpret problems in a variety of situations” (OECD 2007, p. 16). Rindermann (2008, p. 128) maintains there is no important theoretical difference between student achievement and ability tests since they both assess “thinking and knowledge”. Baumert et al. (2009, pp. 3–5) points out that like intelligence tests, reading and mathematical assessments involve reasoning and making logical inferences. Rindermann (2006, 2007, p. 687) concludes there is a strong ‘*g*’-factor, that is the general intelligence factor, in international student assessments, including PISA. Burhan et al.’s (2017) measure of adolescents’ cognitive ability was their PISA test scores. Direct evidence at the individual student level is from the German PISA 2000 study in which students undertaking PISA also sat German cognitive ability tests. Brunner (2008, p. 153) reported correlations of around 0.8 for fluid intelligence with verbal and mathematical test scores.

The measure of *socioeconomic background* used was the OECD’s constructed measure of economic, social and cultural status (ESCS). It was constructed from: the highest international socio-economic index of occupational status of the father or mother; the highest level of education of the father or mother converted into years of schooling; the number of books in the home, and access to educational and cultural resources, which were obtained by asking students whether they had at their home: a desk to study at, a room of their own, a quiet place to study, a computer they can use for school work, educational software, a link to the Internet, their own calculator, classic literature, books of poetry, works of art books to help with their school work, and a dictionary (OECD 2004, p. 307). For these analyses, the ESCS measure was standardized to a mean of zero and a standard deviation of one.

*Year 12 completion* status was based on the constructed variables on respondents’ highest grade of school education at the time of interview. Students who had completed year 12 were assigned a score of one and students that had not, or had not yet, completed year 12 were assigned a score of zero.

*Educational qualifications* were constructed from the derived variable which classified respondents’ highest educational level (for that year) into one of ten categories: VET certificates I, II, III, IV, unknown, advanced vocational diploma, bachelor degree, graduate diploma, higher degree and no post-school qualification. Respondents who had completed an apprenticeship or traineeship were categorized under the appropriate certificate level. From these categories, three one-zero dummy variable measures of educational qualifications were constructed:

- VET certificate III or IV,
- Advanced VET diploma,
- University degree (which includes bachelor degrees and post-graduate qualifications).

The reference category for these dummy variables was not holding any of these qualifications.

Measures of the *proportion of time spent in full*-*time employment* and *proportion of time spent unemployed* were based on the derived variables for full-time employment and unemployment. For each year, it was ascertained if respondents were employed full-time at the time of interview and, had they experienced at least one spell of unemployment during that year (but not necessarily at the time of interview). For each year, data from previous years were used to construct two measures: the proportion of years respondents were employed full-time and the proportion of years they experienced at least one spell of unemployment, independent of study status. For the analyses of employment, unemployment and full-time employment the two proportion measures were lagged by 1 year so that the dependent and independent variables were not based on the same information.

The models include the variable “years” the number of years since 2003, to index improvements in labour market outcomes with time and aging effects.

Appendix Table 7 presents the (weighted) number of cases and means for the analysis variables from 2003 to 2013. In 2003 all respondents were in full-time education so no labour market outcomes could be analysed. For occupational status, earnings and full-time employment, the analyses were restricted to respondents employed and not studying full-time. For employment, the analyses were restricted to respondents not in full-time study. For unemployment, the analyses were restricted to respondents in the labour force.

Appendix Table 7 shows substantial over-time changes in the cohort’s educational qualifications and labour market outcomes. School completion increased dramatically from 17% in 2004 to 71% in 2005 and then plateauing at 80%. For post-school education and training the percentage with a university degree increased from 3% in 2008 to 42% in 2013; from 12 to 27% for VET certificates III or IV; and from 6 to 12% for vocational diplomas. Unemployment declined substantially from 18% in 2005 to < 5% in 2012 and 2013. Average weekly earnings (in 2013 dollars) among employed respondents not studying full-time increased from $554 in 2006 to over $1000 in 2012 and 2013. Appendix Table 8 presents the bivariate correlations between variables in the model. It suggests positive effects of university degrees and to a lesser extent, year 12 completion and combined PISA test score on the six labour market outcomes examined.

### Methods

Generalized estimating equations (GEE) were used to investigate the effects of the predictor variables on the dependent variables over the 10-year period (2004–2013) when respondents were aged between 16/17 and 25/26. The GEE approach enables these relationships to be analyzed for data collected over the entire period rather than for single years. Therefore, the estimates are based on a much larger amount of data and observations lost through attrition of the longitudinal study are included in the analyses.

The generalized estimating equations (GEE) introduced by Liang and Zeger (1986a, b) is a method of analyzing clustered (or correlated) data extending the generalized linear model for non-normal response variables. GEEs have become an important strategy in the analysis of correlated data. Less technical discussions of GEEs are available (Zorn 2001; Ghisletta and Spini 2004).

One important advantage of the GEE approach is a substantial reduction in the amount of missing data. Missing values for which *Y*_*ik*_ are missing whenever *Y*_*ij*_ is missing for all *k *> *j* are called *dropouts*. Otherwise, missing values that occur intermixed with non-missing values are *intermittent* missing values. The GEE approach estimates the working correlation from data containing both types of missing values using the *all available pairs* method, in which all non-missing pairs of data are used in the moment estimators of the working correlation matrix parameters.

GEE analysis is a type of random effects model, but the dependent variable may not be normally distributed. As in the case of generalized linear models link functions are specified to analyze non-normally distributed outcomes. GEE analysis can be understood as a combination of random effects models and generalized linear models. For the analyses of occupational status, no link function was specified so the estimates can be interpreted in the same manner as coefficients obtained from ordinary least squares regression. For the analyses of the earnings, the link function is log because of the highly positive skew of the earnings distributions. The estimated coefficients are interpreted as the percentage change in the dependent variable for a unit change in the independent variable. For the analyses of employment and unemployment the dependent variables are dichotomous and the link function logit, so the coefficients are interpreted as odds ratios which are the exponents of the coefficients.

The correlations of outcome variables among persons in longitudinal studies must be considered. The observations are not statistically independent so the standard errors require adjustment. The within-person correlations were specified as first order autoregressive.

Tables 1, 2, 3, 4, 5 and 6 present the estimates from the analyses. For each labour market outcome three groups of analyses were conducted: for all respondents and separately for young men and young women. The estimates for the two standardized variables—family’s economic, social and cultural status, and combined PISA test score—are the difference on the respective outcome variable, net of other variables in the model, for a one-standard deviation on the predictor variable. The estimates for the dichotomous dummy variables are interpreted as simply the difference on the labour market outcome between a score of one (e.g. being male, having completed year 12, having a university degree) contrasted to a score of zero (e.g. being female, having not completed year 12, not having a university degree). For the proportion measures the coefficients are the difference on the outcome measure between 100% of the time employed full-time (or unemployed) since 2003 and no time spent employed full-time (or unemployed) since 2003. Few observations score one and zero, so it may be better to divide the estimates by 10 which is the estimate for a 10% increase in the proportion of years employed full-time employment or the proportion of years with spells unemployed.

**Table 1:** Effects of Qualifications and Covariates on Occupational Status

	All				Young men				Young women			
Intercept	33.59***		33.46***		31.01***		30.84***		32.90***		32.77***	
Male	− 3.88***		− 3.90***		–		–		–		–	
Parents’ ESCS (std.)	1.39***		1.39***		1.92***		1.94***		0.99**		0.97**	
PISA test score (std.)	2.30***		2.00***		2.83***		2.48***		1.53***		1.25***	
Year 12 completion	3.53***		3.67***		3.29***		3.46***		3.20***		3.33***	
VET certificate III or IV	1.49**		1.49**		1.76*		1.79*		0.92		0.89	
VET diploma	6.20***		6.15***		7.56***		7.52***		4.50***		4.44***	
University degree	20.81***		20.12***		21.38***		20.39***		20.48***		19.94***	
Prop. yrs. FT employed	5.56***		5.44***		2.44		2.23		10.61***		10.55***	
Prop. yrs. unemp. spells	− 1.50		− 1.50		− 1.52		− 1.41		− 2.45*		− 2.53*	
Years since 2003	1.73***		1.72***		1.68***		1.69***		1.76***		1.76***	
Degree by test score	–		1.74*		–		2.09		–		1.58	
N of observations	24,351		24,351		11,913		11,913		12,438		12,438	
N of persons (clusters)	7415		7415		3602		3602		3813		3813	
Clusters with missing data	2372		2372		1149		1149		1223		1223	

Regression coefficients from repeated design analyses. Full-time study observations excluded. Weighted for sample selection and attrition

*ESCS* economic, social and cultural status, *VET* vocational educational and training, *FT* full-time, *prop. yrs. FT employed* proportion of years since 2003 with full time employment, *prop. yrs. unemp. spells* proportion of years since 2003 with any spell of unemployment

* 0.05 > P >  0.01; ** 0.01 > P >  0.001; *** P < 0.001

**Table 2:** Effects of qualifications and covariates on hourly earnings

	All				Young men				Young women			
Intercept	2.87***		2.86***		2.97***		2.97***		2.87***		2.88***	
Male	0.13***		0.13***		–		–		–		–	
Parents’ ESCS (std.)	0.02*		0.02*		0.04**		0.04**		0.00		0.00	
PISA test score (std.)	0.03**		0.03*		0.01		0.01		0.05***		0.07**	
Year 12 completion	− 0.04		− 0.04		− 0.05		− 0.05		− 0.01		− 0.01	
VET certificate III or IV	0.02		0.01		0.03		0.03		− 0.01		− 0.01	
VET diploma	0.02		0.02		0.01		0.01		0.06*		0.04	
University degree	0.09***		0.09***		0.10***		0.09**		0.09***		0.09***	
Prop. yrs. FT employed	0.17***		0.17***		0.25***		0.24***		0.02		0.03	
Prop. yrs. unemp. spells	− 0.18***		− 0.18***		− 0.22***		− 0.22***		− 0.08		− 0.08	
Years since 2003	0.08***		0.08***		0.08***		0.08***		0.08***		0.08***	
Degree by test score	–		0.01		–		0.03		–		− 0.03	
N of observations	22,036		22,036		10,878		10,878		11,158		11,158	
N of persons (clusters)	7415		7415		3602		3602		3813		3813	
Clusters with missing data	3585		3585		1694		1694		1891		1891	

Logged regression coefficients from repeated design analyses. Full-time study observations excluded. Weighted for sample selection and attrition. See Table 1 for abbreviations

* 0.05 > P  >  0.01; ** 0.01 > P > 0.001; *** P < 0.001

**Table 3:** Effects of qualifications and covariates on weekly earnings

	All				Young men				Young women			
Intercept	6.03***		6.03***		6.26***		6.26***		5.98***		5.98***	
Male	0.21***		0.21***		–		–		–		–	
Parents’ ESCS (std.)	0.04***		0.04***		0.04*		0.04**		0.03***		0.03***	
PISA test score (std.)	0.02		0.01		0.02		0.01		0.04***		0.03	
Year 12 completion	0.06		0.06		0.02		0.03		0.13***		0.14***	
VET certificate III or IV	0.03		0.03		0.03		0.03		0.01		0.01	
VET diploma	0.03		0.03		0.00		0.00		0.10***		0.10***	
University degree	0.27***		0.25***		0.24***		0.22***		0.30***		0.29***	
Prop. yrs. FT employed	1.07***		1.06***		1.06***		1.06***		1.01***		1.01***	
Prop. yrs. unemp. spells	− 0.07		− 0.07		− 0.08		− 0.08		− 0.01		− 0.01	
Years since 2003	0.06***		0.06***		0.06***		0.06***		0.05***		0.05***	
Degree by test score	–		0.04*		–		0.05		–		0.04*	
N of observations	22,103		22,103		10,922		10,922		11,181		11,181	
N of persons (clusters)	7415		7415		3602		3602		3813		3813	
Clusters with missing data	3585		3585		1694		1694		1891		1891	

Logged regression coefficients from repeated design analyses. Full-time study observations excluded. Weighted for sample selection and attrition. See Table 1 for abbreviations

* 0.05 > P > 0.01; ** 0.01 > P > 0.001; *** P < 0.001

**Table 4:** Effects of qualifications and covariates on employment

	All				Young men				Young women			
Intercept	1.05***		1.04***		1.08***		1.09***		1.17***		1.15***	
Male	0.14		0.14		–		–		–		–	
Parents’ ESCS (std.)	0.08*		0.08*		0.04		0.04		0.11*		0.11*	
PISA test score (std.)	0.23***		0.22***		0.18***		0.19***		0.28***		0.25***	
Year 12 completion	0.62***		0.63***		0.71***		0.70***		0.58***		0.59***	
VET certificate III or IV	0.19		0.19		0.12		0.12		0.25		0.25	
VET diploma	0.59***		0.59***		0.43*		0.43*		0.68***		0.68***	
University degree	0.94***		0.93***		0.74***		0.78***		1.02***		1.00***	
Prop. yrs. FT employed	2.91***		2.91***		3.02***		3.03***		2.57***		2.58***	
Prop. yrs. unemp. spells	− 0.92***		− 0.93***		− 0.87***		− 0.87***		− 0.96***		− 0.99***	
Years since 2003	− 0.07***		− 0.07***		− 0.01		− 0.01		− 0.10***		− 0.11***	
Degree by test score	–		0.10		–		− 0.14		–		0.28	
N of observations	26,964		26,964		13,058		13,058		13,906		13,906	
N of events (y = 1)	23,935		23,935		11,733		11,733		12,202		12,202	
N of persons (clusters)	7415		7415		3602		3602		3813		3813	
Clusters with missing data	1166		1166		549		549		617		617	

Logit regression coefficients from repeated design analyses. Contrast employed vs not-being-employed. Full-time study observations excluded. Weighted for sample selection and attrition. See Table 1 for abbreviations

* 0.05 > P > 0.01; ** 0.01 > P > 0.001; *** P < 0.001

**Table 5:** Effects of qualifications and covariates on unemployment

	All				Young men				Young women			
Intercept	− 2.22***		− 2.19***		− 1.85***		− 1.87***		− 2.35***		− 2.30***	
Male	0.23*		0.23*		–		–		–		–	
Parents’ ESCS (std.)	− 0.10*		− 0.10*		− 0.11		− 0.11		− 0.10		− 0.10	
PISA test score (std.)	− 0.35***		− 0.32***		− 0.32***		− 0.35***		− 0.37**		− 0.28***	
Year 12 completion	− 0.47***		− 0.48***		− 0.66***		− 0.65***		− 0.29*		− 0.32*	
VET certificate III or IV	− 0.06		− 0.06		0.13		0.14		− 0.25		− 0.24	
VET diploma	− 0.25		− 0.25		− 0.13		− 0.13		− 0.36		− 0.35	
University degree	− 0.56**		− 0.57***		− 0.64**		− 0.66**		− 0.50		− 0.58**	
Prop. yrs. FT employed	− 2.92***		− 2.91***		− 3.11***		− 3.12***		− 2.78***		− 2.76***	
Prop. yrs. unemp. spells	1.51***		1.51***		1.47***		1.48***		1.56***		1.64***	
Years since 2003	− 0.01		− 0.01		0.01		0.01		− 0.02		− 0.02	
Degree by test score	–		− 0.23		–		0.19		–		− 0.62	
N of observations	25,492		25,492		12,541		12,541		12,951		12,951	
N of events (y = 1)	1586		1586		816		816		770		770	
N of persons (clusters)	7264		7264		3533		3533		3731		3731	
Clusters with missing data	1043		1043		501		501		542		542	

Logit regression coefficients from repeated design analyses. Contrast unemployed vs employed. Full-time study observations excluded. Weighted for sample selection and attrition. See Table 1 for abbreviations

* 0.05 > P > 0.01; ** 0.01 > P > 0.001; *** P < 0.001

**Table 6:** Effects of qualifications and covariates on full-time employment

	All				Young men				Young women			
Intercept	− 0.59***		− 0.62***		− 0.02		− 0.05		− 0.70***		− 0.73***	
Male	0.42***		0.42***		–		–		–		–	
Parents’ ESCS (std.)	0.02		0.02		− 0.02		− 0.02		0.05		0.05	
PISA test score (std.)	− 0.01		− 0.06		0.00		− 0.06		0.01		− 0.04	
Year 12 completion	0.34***		0.37***		0.19*		0.22*		0.46***		0.48***	
VET certificate III or IV	0.03		0.03		− 0.01		− 0.01		0.09		0.09	
VET diploma	0.08		0.08		− 0.02		− 0.03		0.21		0.20	
University degree	0.66***		0.56***		0.56***		0.45***		0.70***		0.62***	
Prop. yrs. FT employed	3.76***		3.75***		4.06***		4.03***		3.38***		3.38***	
Prop. yrs. unemp. spells	− 0.16		− 0.16		− 0.39*		− 0.37*		0.05		0.04	
Years since 2003	0.00		0.00		− 0.01		− 0.01		0.01		0.00	
Degree by test score	–		0.28***		–		0.27*		–		0.27**	
N of observations	23,935		23,935		11,733		11,733		12,202		12,202	
N of events (y = 1)	16,649		16,649		8768		8768		7881		7881	
N of persons (clusters)	7005		7005		3414		3414		3591		3591	
Clusters with missing data	840		840		402		402		438		438	

Logit regression coefficients from repeated design analyses. Contrast: employed full-time vs not employed full-time. Full-time study observations excluded. Weighted for sample selection and attrition. See Table 1 for abbreviations

* 0.05 > P > 0.01; ** 0.01 > P > 0.001; *** P < 0.001

The tables include additional information: the number of observations, the number of respondents (clusters) and the number of clusters with missing data. For dichotomous outcomes, the tables also include the number of events, that is the number of observations where the respondent was employed, unemployed or employed full-time.

To examine if the benefits from university degrees for labour market outcomes differ across the achievement continuum, interaction terms were included in the second of each pair of analyses. Aggregation of test scores to low and high achieving groups would be undesirable since it discards useful information (Jaccard and Turrisi 2003, p. 87). The coefficients for the interaction terms are interpreted as the difference in the effect of having a university degree on the respective labour market outcome for a unit difference (a one-standard deviation) in combined PISA test score (from Jaccard and Turrisi 2003, p. 35). In the interaction model, the interpretation of the main effects for university degree and test score are for when the other variable equals zero (Jaccard and Turrisi 2003, p. 24). Since test score has been standardized, these estimates should not differ substantially from the estimates in the initial main effects model. Statistical significance is determined in the same manner as for the main effects (Jaccard and Turrisi 2003, pp. 26, 27).

## Results and discussion

One of more striking findings of these analyses is the strong positive effects for a university degree on these labour market outcomes. Net of other predictor variables and ignoring gender differences for the time being, a university degree increased occupational status by about 20 units on a zero to one-hundred-unit scale, hourly earnings by around 10%, and weekly earnings nearly 30%. A university degree increased the odds of employment relative to not-being-employed, by about 2.5 times; reduced the odds of unemployment relative to employment 1.75 times, and increased the odds of full-time versus not-in full-time employment 1.9 times. These sizable benefits from obtaining a university degree cannot be attributed to ability since the analyses include test scores.

In contrast, the benefits of vocational qualifications are not nearly as positive. A VET certificate III or IV has only weak effects on occupational status which were not statistically significant among young women, had no statistically significant effects on the other outcomes for all respondents, and among men and women.

The higher-level VET diploma fares a little better. A VET diploma has only moderate effects on occupational status increasing occupational status between 4 and 8 units compared to 20 units for university degrees. It shows had no statistically significant effects on hourly and weakly earnings among men but among women increased hourly weekly earnings by about 6%, and by about 10% for weekly earnings. The comparable effects for a university degree were 9 and 30%. A VET diploma had sizable positive effects on employment, more so for young women than young men. It had statistically insignificant effects on both unemployment and full-time employment.

There are notable effects of other factors. Men compared to women tend have lower status jobs but higher earnings (both hourly and weekly). There was no gender difference for simply being employed, but men were more likely than women to be unemployed or working full-time.

Net of educational qualifications, test scores and other factors in the model, socioeconomic background (ESCS) had small effects on occupational status, earnings, employment (only among women); weak and barely statistically significant effects on unemployment and no impact on full-time employment.

Net of educational qualifications and other factors in the model, PISA test scores had only small effects on occupational status, slightly stronger among young men than women. Among young women, a one standard deviation increment in PISA test score increased hourly and weekly earnings by around 5%, but had no effect among young men. Test scores moderately increased the odds of employment: by about 1.2 times (for a one-standard deviation increase) among young men and 1.3 times among young women. Test scores more strongly reduced the odds of unemployment by about 1.4 times (for a one standard deviation difference). Test scores had no significant effects on full-time employment.

Completion of year 12 had positive effects on occupational status, no impact on hourly earnings, increased weekly earnings among women but not men, substantially increased the odds of employment and full-time employment, and reduced the odds of unemployment quite substantially among men.

The proportion of time spent working full-time had moderate effects on occupational status among women, strong effects on hourly earnings among men, and for both sexes strong effects on weekly earnings, unemployment and full-time work. Experience of unemployment had scarring effects on employment and unemployment, and among men on full-time employment. The measure of time (centered around the year 2008) was associated with higher occupational status, higher earnings, and higher levels of employment, but was not associated with unemployment and full-time employment.

The analyses that include interaction effects indicate that the benefits of a university degree do not vary by achievement score. The coefficients for the university degree test score interaction terms were small and not statistically significant for hourly earnings, employment and unemployment. For occupational status, there was a statistically significant interaction effect among all respondents, but when the data is analyzed separately for men and women, the interaction effects were no longer statistically significant. Thus, the most appropriate interpretation is that test scores make no difference to the effects of a university degree on occupational status, at least in this cohort between 2004 and 2013. Even if the statistical tests are ignored, the magnitudes of the interaction effects were small relative to the effects of a university degree. For weekly earnings, there were statistically significant interaction effects among all respondents and among young women, but in both instances, the main effects for PISA test score were not statistically significant. Similarly, for full-time employment there were statistically significant interaction effects, but the effects of PISA test score were negative and not statistically significant in each interaction analysis.

The one instance where there were significant effects for both PISA test score and the interaction term was for occupational status among all respondents (Table 1). However, this analysis still shows that lower achievers benefit more from a university degree than from a VET diploma. The returns in occupational status for a university degree among respondents scoring one standard deviation below the mean is 18.4 (20.1–1.74) considerably higher than that for a VET diploma (6.2). Even for the small group of respondents with PISA test scores two standard deviations below the mean, the returns to occupational status from a university degree are still considerably higher (16.6) than that for a VET diploma.

## Conclusions

Logic suggests that university degrees are not as valuable in the labour market for those who without expansion of the university sector would not have been admitted to university. However, this research has found that for these six labour market outcomes, the benefits of university degrees are *not* weaker for lower achieving students. These analyses are for completed degrees. Students with low university entrance scores (which is highly correlated with achievement) are much more likely not to complete their university course (Norton and Cherastidtham 2018) so would not enjoy the labour market benefits of completed degrees. On the other hand, there are sizable positive effects for just enrolling in university courses, at least for wages (Hérault and Zakirova 2015).

These analyses are confined to young people up until age 25 or 26. At older ages, there may be stronger differences between high and low achievers in the labour market returns to university degrees. There are small but persistent effects of ability, measured by test scores during adolescence, on adult labour market outcomes, even when considering educational attainment and in some studies experience in the labour market (Warren et al. 2002; Marks 2014, pp. 99–106). The interactions between university degrees and achievement should theoretically increase as the cohort ages since those in high prestige courses that lead to high status and high earning occupations (e.g. medicine, law or post-graduate courses) would have had graduated and progressed from entry level wages, and other graduates with high achievement scores would have had more time to establish themselves in the labour market. However, employers do not base hiring and promotion decisions on test scores, but on performance in the labour market and discernable labour market characteristics, which depending on the context may or may not be correlated with test scores.

The possibility of stronger university degree test score interactions among older cohorts is irrelevant to the policy question of whether expansion of university education is desirable for academically weaker youths making the transition from full-time education to the labour market. These analyses suggest that overall, low achievers are better off graduating from university than obtaining a vocational certificate or diploma. Therefore, this study supports the present policy position advocated by the major political parties. However, the expansion of university education cannot be understood as a panacea for the youth labour market. There are limits to further expansion of university education both in terms of public costs and the capacity of the graduate labour market to absorb new entrants, especially during economic downturns.

It is clear from these analyses that a university degree has substantial positive effects on early labour market outcomes, at least for this cohort, which cannot be attributed to socioeconomic background, academic ability, or completion of year 12. Vocational diplomas are much less beneficial and vocational certificates confer no, or only weak, positive effects. The analyses presented here reiterate the importance of a ‘good start’ in the labour market. Experience of full-time employment is important to most of these labour market outcomes and spells of unemployment have scarring effects not just on future bouts of unemployment, but also on hourly earnings and employment especially among young men. Policies need to ensure that non-university bound youth can easily obtain full-time work and not become unemployed, and this should be a policy priority over promoting vocational education and training.

## Appendix

See Tables 7 and 8.

**Table 7:** Univariate Statistics for Time Variant Variables

Variable	2003		2004		2005		2006		2007		2008		2009		2010		2011		2012		2013	
	N	Mean	N	Mean	N	Mean	N	Mean	N	Mean	N	Mean	N	Mean	N	Mean	N	Mean	N	Mean	N	Mean
FT study	10,370	1.00	9378	0.90	8691	0.81	7721	0.56	6658	0.52	6074	0.50	5475	0.38	4903	0.26	4429	0.20	3945	0.14	3741	0.12
Male	10,370	0.51	9378	0.51	8691	0.51	7721	0.51	6658	0.50	6074	0.50	5475	0.50	4903	0.50	4429	0.50	3945	0.50	3741	0.50
Parents’ ESCS (std.)	10,307	− 0.03	9323	− 0.02	8644	− 0.02	7686	− 0.02	6629	− 0.02	6049	− 0.02	5456	− 0.03	4885	− 0.02	4414	− 0.02	3935	− 0.02	3732	− 0.01
PISA test score (std.)	10,370	− 0.05	9378	− 0.05	8691	− 0.05	7721	− 0.06	6658	− 0.06	6074	− 0.06	5475	− 0.06	4903	− 0.07	4429	− 0.06	3945	− 0.05	3741	− 0.05
Year 12 completion	10,370	0.00	9378	0.17	8691	0.71	7721	0.77	6658	0.78	6074	0.78	5475	0.79	4903	0.79	4429	0.80	3945	0.80	3741	0.80
VET certificate III or IV	10,370	0.00	9378	0.00	8691	0.01	7721	0.05	6658	0.09	6074	0.12	5475	0.15	4903	0.17	4429	0.18	3945	0.18	3741	0.27
VET diploma	10,370	0.00	9378	0.00	8691	0.00	7721	0.01	6658	0.05	6074	0.06	5475	0.08	4903	0.08	4429	0.09	3945	0.10	3741	0.12
University degree	10,370	0.00	9378	0.00	8691	0.00	7721	0.00	6658	0.01	6074	0.03	5475	0.13	4903	0.25	4429	0.32	3945	0.38	3741	0.42
Prop. yrs. FT employed	10,370	0.02	9378	0.05	8691	0.08	7721	0.14	6658	0.20	6074	0.23	5475	0.26	4903	0.29	4429	0.32	3945	0.35	3741	0.38
Prop. yrs. unemp. spells	10,370	0.00	9378	0.16	8691	0.19	7721	0.22	6658	0.22	6074	0.21	5475	0.21	4903	0.21	4429	0.21	3945	0.20	3741	0.19
Occupational status (SEI)			593	19.78	1227	20.28	2751	33.51	2548	36.95	2400	38.82	2759	42.31	3082	46.39	3140	50.21	3017	52.44	2936	53.64
Hourly earnings			526	10.04	1111	12.79	2505	14.63	2332	17.44	2161	19.55	2534	21.83	2805	23.28	2848	25.37	2592	27.42	2710	29.61
Weekly earnings			530	361	1113	230	2508	554	2337	695	2170	768	2541	839	2820	914	2856	983	2594	1087	2721	1141
Employed			904	0.67	1648	0.75	3295	0.84	2946	0.86	2747	0.87	3157	0.87	3472	0.89	3471	0.89	3335	0.91	3267	0.90
Unemployed			796	0.24	1501	0.18	3083	0.11	2770	0.08	2597	0.08	3000	0.08	3302	0.06	3311	0.06	3181	0.03	3126	0.04
Employed FT			612	0.41	1255	0.47	2773	0.53	2580	0.63	2423	0.71	2806	0.68	3138	0.73	3178	0.77	3089	0.79	3007	0.78

Weighted for sample selection and attrition

Ns for occupational status, earnings and employed full-time = employed and not in full-time study

N for employed = employed, unemployed and not in the labour force, and not in full-time study

N for unemployed = employed and unemployed, and not in full-time study

All other Ns = number of respondents with completed interviews for that year

Earnings adjusted to 2013 dollars

**Table 8:** Bivariate correlations of independent and dependent variables

	Male	ESCS	Test score	Year 12	VET cert. III/IV	VET dipl	Degree	Prop. FT Empl	Prop unem	Years	SEI	Hour earn	Week earn	Emp	Unemp
Parents’ ESCS (std.)	0.01														
PISA test score (std.)	− 0.03	0.41													
Year 12 completion	− 0.05	0.11	0.21												
VET certificate III or IV	− 0.02	− 0.06	− 0.09	0.06											
VET diploma	− 0.02	0.00	− 0.06	0.11	− 0.06										
University degree	− 0.07	0.11	0.18	0.23	− 0.10	− 0.07									
Prop. yrs. FT employed	0.18	− 0.11	− 0.18	0.02	0.29	0.08	0.02								
Prop. yrs. unemp. spells	0.01	− 0.08	− 0.15	− 0.02	0.01	0.01	− 0.03	− 0.12							
Years since 2003	− 0.01	0.00	− 0.00	0.48	0.29	0.20	0.44	0.47	0.04						
Occupational status (SEI)	− 0.07	0.12	0.18	0.33	0.07	0.11	0.51	0.17	− 0.00	0.57					
Hourly earnings (log)	0.05	0.05	0.06	0.25	0.17	0.12	0.29	0.27	0.01	0.61	0.46				
Weekly earnings (log)	0.14	− 0.06	− 0.10	0.22	0.22	0.12	0.27	0.62	0.05	0.66	0.46	0.71			
Employed	− 0.01	0.03	0.02	0.18	0.10	0.07	0.12	0.34	− 0.21	0.29	^a^	^a^	^a^		
Unemployed	0.01	− 0.05	− 0.08	− 0.01	− 0.02	− 0.01	− 0.04	− 0.14	0.39	− 0.04	^b^	^b^	^b^	− 0.47	
Employed full-time	0.13	− 0.07	− 0.11	0.14	0.19	0.09	0.19	0.79	− 0.08	0.46	0.32	0.25	0.70	0.42	− 0.19

Weighted person-year data. Employed is of all non-full-time students. Unemployed is of labour force participants. Employed full-time is of employed

^a^Not applicable because contrast group includes respondents not employed

^b^Not applicable because includes respondents not employed
